# Clustering and separation of hydrophobic nanoparticles in lipid bilayer explained by membrane mechanics

**DOI:** 10.1038/s41598-018-28965-y

**Published:** 2018-07-17

**Authors:** Matej Daniel, Jitka Řezníčková, Milan Handl, Aleš Iglič, Veronika Kralj-Iglič

**Affiliations:** 1 Department of Mechanics, Biomechanics and Mechatronics, Faculty of Mechanical Engineering, Czech Technical University in Prague, 16600 Prague 6, Czech Republic; 2 University Hospital Motol, Charles University in Prague, 150 06 Prague 5, Czech Republic; 3 Dubai Healthcare City, Dubai, UAE; 4 Laboratory of Biophysics, Faculty of Electrical Engineering, University of Ljubljana, SI-1000 Ljubljana, Slovenia; 5 Laboratory of Clinical Biophysics, Faculty of Health Sciences, University of Ljubljana, SI-1000 Ljubljana, Slovenia

## Abstract

Small hydrophobic gold nanoparticles with diameter lower than the membrane thickness can form clusters or uniformly distribute within the hydrophobic core of the bilayer. The coexistence of two stable phases (clustered and dispersed) indicates the energy barrier between nanoparticles. We calculated the distance dependence of the membrane-mediated interaction between two adjacent nanoparticles. In our model we consider two deformation modes: the monolayer bending and the hydroxycarbon chain stretching. Existence of an energy barrier between the clustered and the separated state of nanoparticles was predicted. Variation analysis of the membrane mechanical parameters revealed that the energy barrier between two membrane embedded nanoparticles is mainly the consequence of the bending deformation and not change of the thickness of the bilayer in the vicinity of nanoparticles. It is shown, that the forces between the nanoparticles embedded in the biological membrane could be either attractive or repulsive, depending on the mutual distance between them.

## Introduction

Unique electronic, optical, catalytic, and magnetic properties of nanoparticles (NPs) make them extremely interesting for a variety of biomedical applications^1–4^. When interacting with cells, the first barrier that NPs need to encounter is the plasma membrane. Multiple computational and experimental studies have previously explored the interaction between the membrane and NPs^1,5–11^ showing possibility of transmembrane trafficking^12^, poration induced by NPs^13^, encapsulation of NPs^14,15^, change in membrane fluidity^14,16^, NP ordering^9^ and clustering mediated by the membrane^17^. Accumulation of NPs in the membrane is driven by the NP shape, size, stiffness, and the nature of its interaction with the membrane^5,11,14,17–20^.

The NP interacting with a membrane perturbs the membrane causing short-range and long-range forces^6,9,21^. The biomembrane transmits the forces between spatially separated NPs which is denoted as the membrane-mediated interaction^22^. Several possible mechanisms of the membrane-mediated NPs interactions have been proposed. The driving force of the self-assembly of NPs could be ascribed to the hydrophobic mismatch as the membrane thickness is altered in the vicinity of the embedded NP^23–25^. It has been reported for membrane inclusions, that if two adjacent inclusions alter the membrane thickness in the same manner, they will attract each other, whereas if one inclusion thins and the other thickens the bilayer, the inclusions will repel each other^26^. Furthermore, the NP’s intrinsic curvature may affect the membrane curvature and generate attractive forces between NPs, if the adjacent inclusions have the opposite intrinsic curvature^27,28^. Even in the case of the NPs that match the thickness and the curvature of the membrane, the NPs could be attracted due to the long-range Casimir-like forces in the fluctuating membrane^29^ or due to the short range depletion attraction forces^24,30^.

**Figure 1:**
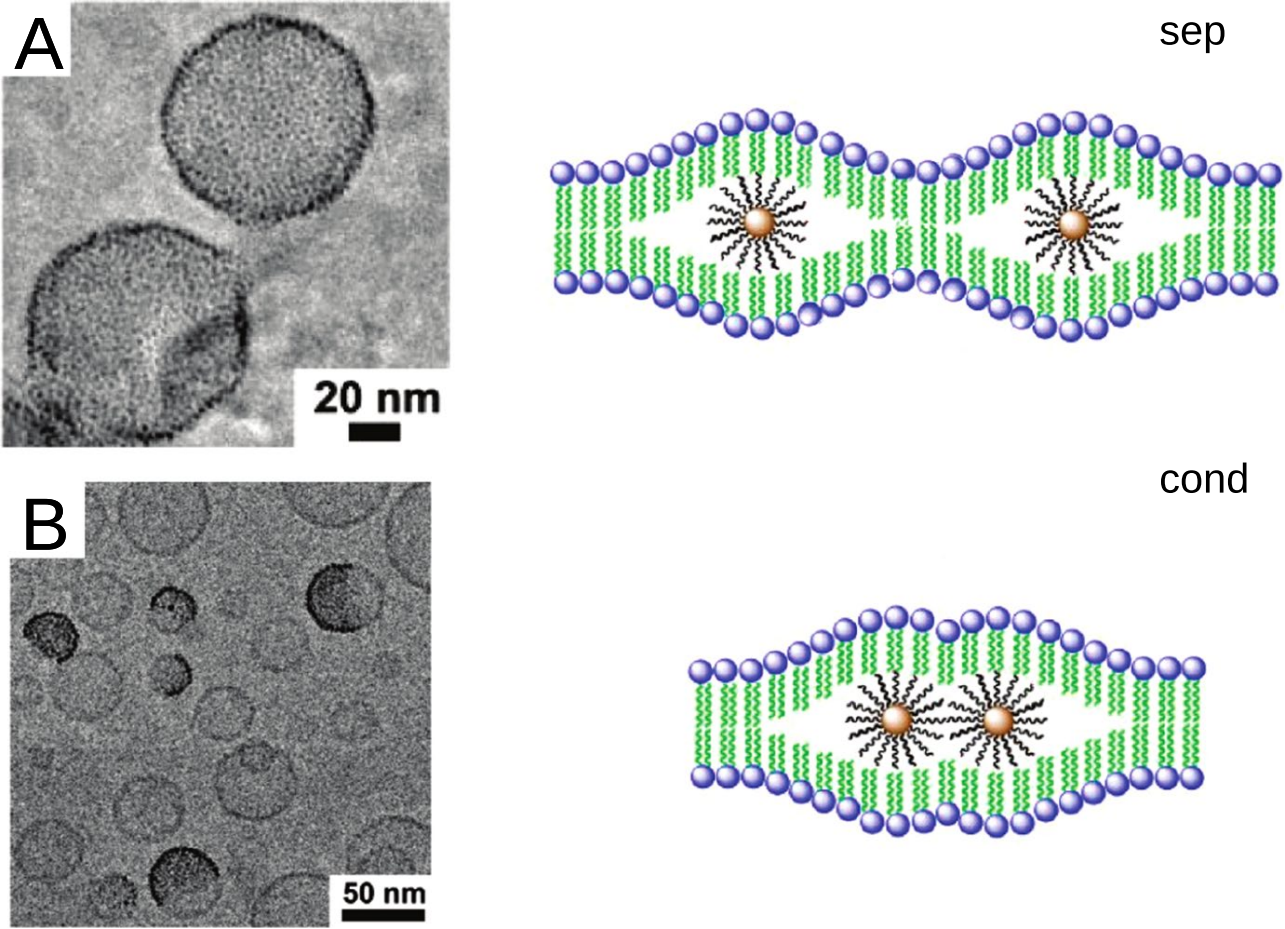
(**A**) Vesicles prepared by coextrusion of lipids and nanoparticles; NPs in the membrane are separated (sep). (**B**) Janus-type of NP-vesicle hybrids prepared by loading of NPs into pre-prepared vesicles; NPs in the membrane are condensed (cond). Adapted with permission from Rasch *et al*., 2010. Copyright 2010 American Chemical Society.

Small NPs (diameter < 10 nm) are more likely to form membrane channels^31^, while the larger NPs (diameter > 10 nm) usually penetrate into cells through membrane wrapping and internalisation^32^. Within this study, we consider hydrophobic nanoparticles with diameter smaller than the membrane thickness, that can penetrate the outter membrane layer and accumulate in bilayer cores^10,33^. We addressed the specific phenomena which show that the method of loading of 2 nm-sized gold NPs into lipid vesicles affects the NPs distribution within the biological membrane^34^. If the NPs and phosphatidylcholine lipids are coextruded, the NPs form a dense monolayer in the hydrophobic core of the vesicle membrane (Fig. 1A). In contrast, vesicles which are formed by extrusion and then dialyzed in the presence of NPs dispersed with detergent contain a membrane region with NPs and a membrane region without NPs (Fig. 1B). In these vesicles, so-called the Janus-like vesicles, the NPs aggregate and form clusters in the membrane regions that are rich with NPs.

The two stable states of NPs in the vesicle membrane may be explained by the existence of an energy barrier between aggregated and separated NPs mediated by a biomembrane^34^. The aim of this study is to test this hypothesis by estimation of the energy of a biomembrane induced by redistribution of NPs.

**Figure 2:**
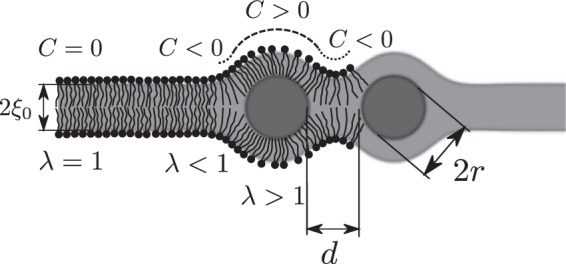
Geometrical model of the lipid bilayer with two embedded NPs of radius *r* at distance *d*. The thickness of the phospholipid hydroxycarbon tail region in the unperturbed planar lipid bilayer is denoted by *ξ*_0_. NPs induce changes in the local curvature of the bilayer leaflets *C* and the change of the membrane thickness due to deformation of hydroxycarbon tails *λ* = *ξ/ξ*_0_.

It was shown in the past that considering the membrane as continuum elastic medium may well explain interactions between membrane inclusions like transmembrane proteins^24,35,36^. Two key modes of membrane deformation caused by hydrophobic inclusion are the hydrophobic mismatch causing deformation of hydroxycarbon chains (stretching/compression) and the membrane bending of both membrane lipid bilayers^10^. Within this study, two rigid NPs of diameter *r* separated by the distance *d* are considered (Fig. 2). The local deformation of the membrane by the inclusions is then analysed by the variation of the membrane elastic energy which is increased by intercalation of NPs into hydrophobic moiety of membrane^28^. The local equilibrium shape of the membrane is determined as a shape with minimal elastic energy of the membrane in the deformed state. The variations of the intrinsic curvature *C*_0_, the bending constant *κ*_*b*_, and the compression-expansion constant *κ*_*c*_ are performed to study the effect of membrane properties on the elastic energy of membrane with the NP inclusions. The range of the membrane elastic parameters adopted in simulations is shown in Table 1.

**Table 1:** Geometrical and mechanical parameters of the NP and phosphatidylcholine membrane monolayer. Bold numbers denote the default values of parameters.

Parameter		Value	Reference
NP size	*r* [nm]	1, **2**, 3, 4	^10^
intrinsic curvature	*C*_0_ [nm^−1^]	−0.1, **−0.2**, −0.3	^42^
bending moduls	*κ*_*b*_ [*k*_*b*_*T*]	5, **10**, 15	^53^
compression modulus	*κ*_*c*_ [*k*_*b*_*T* nm^−2^]	30, **45**, 60	^54^
monolayer thickness	*ξ*_0_ [nm]	**1.47**	^55^
lipid area	*a*_0_ [Å^2^]	**72.4**	^56, 57^

## Results

**Figure 3:**
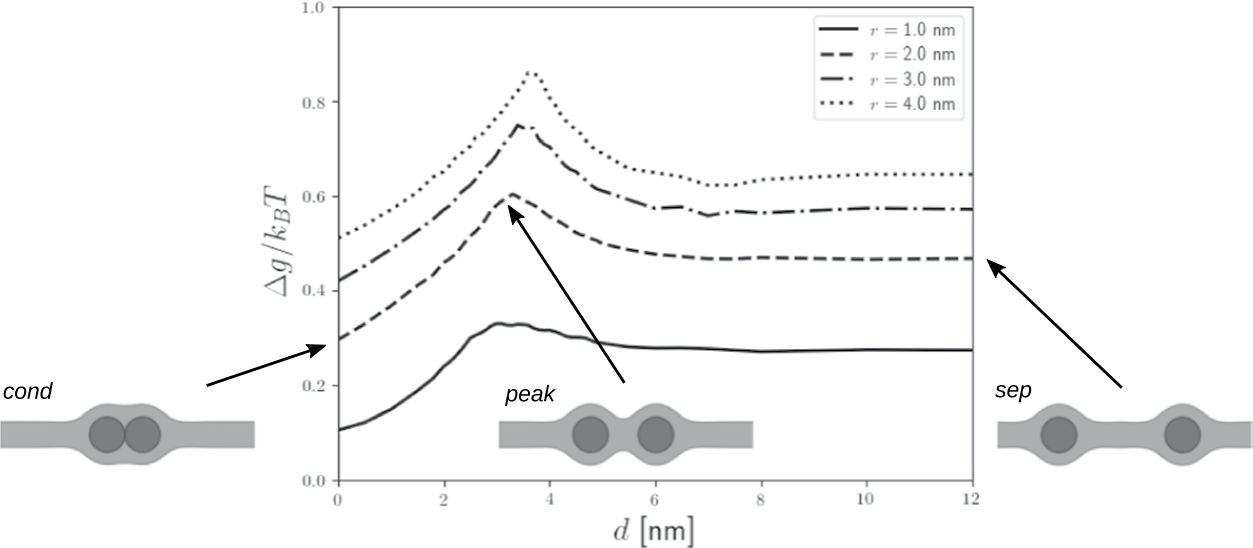
The membrane elastic energy per lipid molecule in the presence of nanoparticles *g* considering the planar lipid bilayer energy *g*_planar_ as reference (Δ*g* = *g* − *g*_planar_) in dependence on the distance between the nanoparticles *d* for various sizes of nanoparticle.

Figure 3 shows the dependence of the calculated membrane elastic energy on the distance between two NPs and the corresponding membrane elastic energy for various values of NP diameter. It can be seen in Fig. 3 that the condensed state of NPs is energetically more favorable than the separated state. Small nanoparticles require less elastic energy to embed into the lipid bilayer, in agreement with Wi *et al*.^10^.

Further, our simulations confirms the hypothesis of Rasch *et al*.^34^, that there exists an elastic energy barrier between disperse and aggregate state of NPs embedded in the biological membrane (Fig. 3).

**Figure 4:**
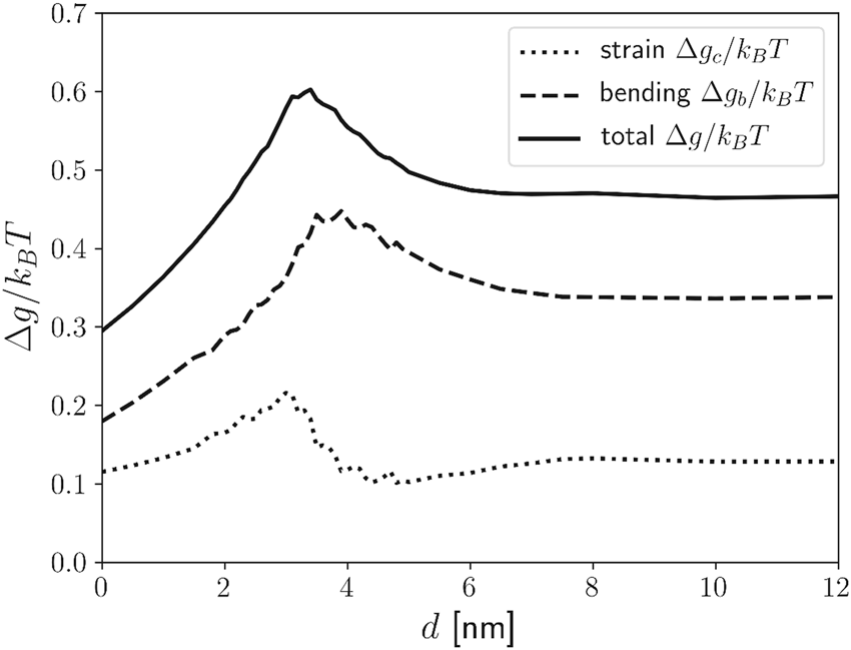
Contribution of stretching (Δ*g*_*c*_) and bending energy (Δ*g*_*b*_) to the relative elastic energy per lipid molecule (Δ*g*) in the presence of nanoparticles (*r* = 2 nm) as a function of the distance between the nanoparticles. Energies are expressed by taking the planar lipid bilayer elastic energy as reference.

**Figure 5:**
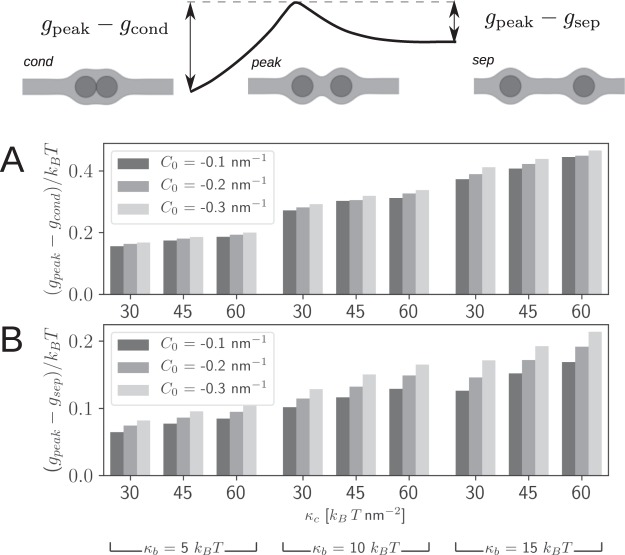
The height of the energy barrier with respect to (**A**) the condensed and (**B**) the separated phase of NPs calculated for different values of membrane parameters *C*_0_, *κ*_*b*_, and *κ*_*c*_.

The bending contribution to the energy per lipid molecules is higher than the stretching contribution of hydroxycarbon tails (Fig. 4). Figure 5 shows the height of the energy barrier in condensed (Fig. 5A) and separated (Fig. 5B) state of NPs, calculated for different values of membrane parameters *C*_0_, *κ*_*b*_, and *κ*_*c*_. It is shown that the membrane bending rigidity *κ*_*b*_ has the largest effect on the magnitude of the energy barrier. The higher the values of intrinsic curvature *C*_0_, membrane bending *κ*_*b*_ and compression modulus *κ*_*c*_, the higher the energy barrier that the nanoparticles must overcome to separate (Fig. 5A) or to cluster themselves (Fig. 5B). The height of the elastic energy barrier is generally lower for transition from separated to clustered phase than in the opposite direction–from clustered into separated state.

## Discussion

It was experimentally observed that the lateral distribution of hydrophobic NPs in a lipid membrane vesicle depends on the method of loading of NPs into the lipid vesicle^34^. If the NPs are incorporated into the membrane of the already existing lipid vesicle by the dialysis process, they form NP clusters in the membrane indicating that the NP clustering is energetically favorable^37^. However, if the membrane is self-assembled with NPs, it was observed that NPs are distributed uniformly in the membrane^34^.

Our results could explain the aggregation of NPs in the lipid bilayer prepared by dialysis observed by Bonnaud *et al*.^37^ and Rasch *et al*.^34^. Insertion of the first NP disturbs the membrane locally. It is energetically more favorable for the next intercalated particles to be clustered with the other membrane-inserted particle (Fig. 5A) than to be dispersed. If the clustered nanoparticles are separated, e.g. by small thermal fluctuations, the membrane-mediated interaction attract them together. However, if the membrane is self-assembled together with NPs, the NPs are embedded in the membrane at a distance larger than the one corresponding to the membrane elastic energy peak in Fig. 3. In this case, membrane-mediated interactions cause repulsive forces between NPs (Fig. 3) and NPs remain unclustered.

In general, the membrane-mediated interactions may induce either attractive or repulsive forces between the biomembrane inclusions^23–27,29^. Considering the gradient of membrane elastic energy in Figs 3 and 4, we may conclude, that both cases are possible for small hydrophobic NPs embedded in hydrophobic core of membrane, depending on the mutual distance between NPs.

In agreement with our model, the recently published coarse-grained molecular dynamics study^9^ also predicted the existence of energy barrier between two anionic ligand-coated NPs embedded in a cholesterol-free lipid membrane. The peak energy of the energy barrier was observed at roughly 8 nm distance between the NPs of diameter 3 nm. It corresponds to the distance *d* = 5 nm as defined in Fig. 2. Our simulations predict energy barrier peak at the distance *d* equal to 4 nm. However, the previous study^9^ was focused on NPs coated with amphiphilic ligands and they are therefore not fully included in the hydrophobic core of the lipid bilayer. Also, the particles in this study^9^ were negatively charged (ammonic), therefore they repel each other electrostatively.

Rasch *et al*., 2010 hypothesized that the energy barrier appears mainly due to the phospholipid tail stretching deformation. Our simulations show that instead of the deformation of membrane hydrophobic core, bending of the monolayer is the decisive factor influencing the membrane elastic energy barrier between two neighbouring membrane embedded particles (Fig. 4). Increase of the bending stiffness of the phosholipid monolayers considerably increases the bilayer energy associated with NP insertion in the membrane and the height of the energy barrier between the condensed (Fig. 5A) and the dispersed (Fig. 5B) state of NPs.

The present study employs the number of assumptions to intentionally keep the model simple. Our main goal was to qualitatively illustrate the membrane elasticity-driven interactions between NPs in the biomembrane. We have adopted a continuum approach although the studied length scale is at molecular dimensions. Describing the biomembrane as elastic continuum is certainly an approximation. However, studies of protein channels^38^ show that hydrophobic matching and membrane-mediated interactions can be well understood also within simple elasticity theory.

The model could be further upgraded by considering the lipid tilt^24,36,39,40^. May *et al*.^36^ showed that the lipid tilt modulus has two energy contributions. The first one is included in the present model as the stretching of the hydrocarbon chains and reflects the loss of chain conformational freedom. The second contribution that was not considered in this work is the entropic contribution resulting from the constraints imposed by the tilt deformation on the fluctuations of the hydroxycarbon chain. We may assume that the entropic contribution is less pronounced in the case of a fully embedded NP than in the case of conformational restrictions that flexible hydroxycarbon chains experience in the vicinity of a rigid transmembrane inclusion^24^.

The model assumes that there are no constraints on the distribution of phospholipids between NPs and planar biomembrane. This assumption corresponds to strong hydrophobic interaction between the NP and hydroxycarbon chains of membrane lipids. In the study of Rasch *et al*., 2010, such strong hydrophobic interaction is mediated by dodecanethiol coating of gold NPs In the case of weaker interaction, the larger NPs might induce membrane poration^33^, that is not considered within this study.

Our model also does not explicitly take into account the increase of the disorder of lipids in the vicinity of the NP^25^. This effect might be important in the lipid bilayer containing cholesterol, where the cholesterol content was shown to decrease near NP^25^. It was indicated that purely hydrophobic NPs accumulate at the interface between the cholesterol-rich, ordered domains and cholesterol-lean, liquid domains to reduce the net interfacial free energy^9^. The interfacial energy minimization was also proposed for protein self-assembly mediated by disordered domains around protein inclusions within ordered lipid bilayers^41^. The presence of cholesterol may also contribute to the depletion forces^24^ between the NPs. These additional attractive forces may be responsible for NP clustering observed in the presence of cholesterol in the membrane^9^.

Our simulations were performed for phosphatidylcholine lipids with negative intrinsic curvature *C*_0_ (Table 1). The effect of variation of the intrinsic curvature on the membrane energy is minor as contributions of the positive and the negative curvature regions to elastic membrane energy are canceled (Fig. 2). Therefore, we may expect the existence of energy barrier between the condensed and the separated state of NPs also for membranes composed of the lipids with positive intrinsic curvature.

Within this study a membrane composed of single lipid sort was considered. The lipid mixture with various intrinsic curvatures^42^ could influence NP-biomembrane interaction. We may expect also lipid sorting at the highly curved NP surface^43^. In this case, the Gaussian energy term should be included in equation (1) and entropic contribution^44–46^ due to non-homogenous lateral distribution of lipids should be considered.

The presented study is based on two-dimensional analysis by taking the second principal curvature in the membrane to be zero. In three-dimensions, the non-zero second principal curvature should be taken into account. The Gaussian bending energy could also be considered constant in three-dimensions if the studied membrane area is the part of closed vesicle and the presence of NPs will not influence the membrane shape outside the studied region. Considering the second principal curvature in three dimensions would likely change the values of the elastic energy, but it would not influence the main conclusions about the role of the energy barrier between the NPs in biomembrane, preventing either their clustering or disassembling of already formed clusters.

## Methods

The NP embedded within the lipid bilayer hydrophobic region induces perturbation in bilayer thickness and bends both bilayer leaflets (Fig. 2). The local shape of the lipid bilayer wrapping around the NP is governed by the interplay between the lipid tails’ stretching energy *G*_*c*_ and the bending energy of both monolayers *G*_*b*_. The local bending energy can be expressed as deviation of the membrane local curvature *C* from initrinsic membrane curvature *C*_0_^47^, while the transversal stretching energy is considered as the deformation of the hydrophobic core^35,48^.

Within this study, the problem of NP interaction is reduced to two-dimensions, as shown in Fig. 2. Two rigid NPs of diameter *r* separated by the distance *d* are considered. The average energy per lipid molecule can be expressed as

1 $$g=\frac{1}{2}\frac{{a}_{0}}{l}{\int }_{A}{\kappa }_{b}{(C-{C}_{0})}^{2}+{\kappa }_{c}{(\frac{\xi -{\xi }_{0}}{{\xi }_{0}})}^{2}{\rm{d}}l$$

where *κ*_*b*_ and *κ*_*c*_ are the bending and the compression-expansion constants of the lipid layers, respectively, *ξ* is the thickness of the deformed monolayer in the vicinity of the NP, *ξ*_0_ is the length of the lipid tails of an unperturbed monoloayer, and *a*_0_ is the area per lipid mocule. The integration is performed over the contour length *l* of the monolayers at the hydrophobic/water interface.

The optimal shape is determined by the minimum of the average elastic energy per lipid in the presence of nanoparticle *g* with respect to its energy in the planar lipid bilayer *g*_planar_ (Δ*g* = *g* − *g*_planar_). The energy per lipid molecule in planar bilayer can be expressed by taking C = 0 and *ξ* = *ξ*_0_ in Eq. (1).

The configuration of the system corresponding to its minimal elastic energy was obtained by using the custom written optimization program employing basin hopping method^49^. The membrane contour is discretized and each step of the random perturbation is followed by the local minimization of the membrane elastic energy using the sequential quadratic programming^50^. The simulated annealing acceptance test based on standard Monte Carlo was used^51^. To reduce computational demands, one quarter of the geometry is solved considering symmetry about bilayer midplane and midplane between NPs (Fig. 2). The predictions of our model were verified by comparison with the results of an analytical model of Sub Wi *et al*.^10^ for a single NP embedded in the membrane. The membrane element size was chosen on the basis of the mesh convergence test. The lipid parameters chosen for the simulation are shown in Table 1. Simulations are based on default values of parameters, if not stated otherwise. The range of the NP size is taken after Gopalakrishnan *et al*.^52^, who observed no NPs larger than 4 nm embedded in the lipid bilayer. To study the effect of membrane properties on the elastic energy of membrane with the NP inclusion, the variations of the intrinsic curvature *C*_0_, the bending *κ*_*b*_, and the compression-expansion constants *κ*_*c*_ are performed. The range of the membrane elastic parameters adopted in simmulations is shown in Table 1.

### Data availability

All data generated during this study are included in this manuscript. The code of the computational framework in Python is available from the corresponding author on reasonable request.
